# Supplementary material for: Hotspot mutations and ColE1 plasmids contribute to the fitness of Salmonella Heidelberg in poultry litter
Source: PLoS One. 2018 Aug 31;13(8):e0202286. doi: 10.1371/journal.pone.0202286 (PMC6118388; doi:10.1371/journal.pone.0202286)
Supplement: S1 Text — (DOCX) [file pone.0202286.s001.docx]

**SUPPLEMENTARY MATERIALS AND METHODS**

**DNA sequencing and bioinformatics.** Plasmids were classified into MOB families, using methods described previously (1), which were implemented using scripts available at <https://github.com/AlexOrlek/MOBtyping/releases/tag/v2.0>. Briefly, relaxase proteins representing MOB families (MOBC, MOBF, MOBH, MOBP, MOBQ, MOBV) were queried against a plasmid database, using PSI-BLAST searches (2) run to ≤14 iterations. The plasmid database included contigs from samples in this study, along with a broader collection of complete reference plasmids, included to ensure relaxase sequence diversity was well-represented (3). To investigate the topology of plasmid contigs - whether it is circular or linear, and if linear, whether it is connected to other contigs, we used a custom script to inspect the path in the SPAdes FASTG assembly graph file, that corresponded to the plasmid contig identified in the FASTA file. The contigs.paths file was used to determine correspondence between FASTA contigs and FASTG edges. Plasmid contigs were assigned as ‘complete circular’; ‘complete non-circular’, i.e. linear but not connected to other contigs; and ‘non-complete’, i.e. linear and connected to other contigs. Since linear Gammaproteobacterial plasmids are rare (3), complete non-circular plasmids may well represent plasmid fragments, isolated in the assembly graph due to poor recovery of adjacent sequence content. In cases where a contig corresponded to multiple edges in the FASTG, which were subsequently joined through repeat resolution (4), the assembly graph was visualised using Bandage (5); however, since SPAdes assemblies are not maintained in graph format after repeat resolution (6), circularity could be ruled out in cases where the path was incomplete, but never ruled in.

**Extraction of 23S-5S ITS sequence**. The internal transcribed spacer (ITS) DNA sequence situated between the large ribosomal (23S rRNA) and small ribosomal (5S rRNA) was identified by mapping whole genome sequences of *S*. Heidelberg genomes to *S*. Heidelberg reference genome (CP016573). This ITS region is between loci position 4035504 – 4035584 of the reference genome. After mapping, a consensus sequence was determined for each strain/isolate using Geneious v. 11.1 and aligned with MAFFT (7). Phylogenetic tree was produced from aligned DNA sequences using the maximum likelihood method implemented in MEGA v. 7 (8) using the Hasegawa-Kishino-Yano model and a rapid bootstrapping run to generate 1000 replicates.

**Antibiotic susceptibility testing.** Disk diffusion testing was performed with aminoglycoside and fosfomycin discs on strains recovered at Day 0 and Day 14. Single colonies were isolated on sheep blood agar (Remel brand, Lenexa, KS) and resuspended in 2 ml 1X PBS after 24 h of incubation at 37 ^o^C. The turbidity of the resuspension was compared to a MacFarland standard of 0.5 to provide an approximate cell density of 1.5x10^8^ ml^-1^. Within 15 minutes of preparation, a sterile cotton swab was dipped into the test solution and streaked onto Mueller Hinton (MH) agar in 100 X 15 mm petri dish. Afterwards, sterile forceps were used to transfer antibiotic disks onto streaked MH plates; three disks to a plate per strain. The following antibiotic disks and concentrations were used for the susceptibility testing: tobramycin (10 µg), gentamicin (10 µg), kanamycin (30 µg), neomycin (30 µg), netilmicin (30 µg), and fosfomycin (200 µg) with glucose –6-phosphate. Aminoglycoside discs were obtained from Remel (Remel Inc. Lenexa, KS) while fosfomycin were from Becton Dickinson, Hong Kong (BBL Sensi-Disc). Gentamicin, fosfomycin, and netilmicin discs were placed equidistant from each other on a plate while tobramycin, kanamycin, and neomycin were placed together. Disc diffusion plates were incubated at 37 ^o^C for 24 h. The zones of inhibition surrounding each antibiotic disk was measured with a ruler by three independent laboratory technicians; and reported as an average (S3 File). The zones were also measured after 48 hours with no significant differences reported. *Pseudomonas aeruginosa* (ATCC 27853*)* and *Escherichia coli* (ATCC 25922) was used as a positive and negative control respectively.

**Chemical analyses.** The pH of recovered PL eluate was determined with a portable Orion Star A series pH meter (Thermo Scientific, Rockwood, TN). For nutrient analyses, saved eluate were removed from -80 ^o^C and allowed to thaw completely. Once thawed, samples were centrifuged for five minutes at 4,600 x *g*. Samples were then filter sterilized through a 0.45 μm syringe filter (Thermo Scientific, Rockwood, TN). Following filtration, samples were diluted 10-fold in autoclaved nanopure water. Total C in the extracts was analyzed using a Shimadzu TOC-L instrument (Shimadzu Scientific Instruments, Columbia, MD) using Standards Methods 5310B for high-temperature catalytic oxidation. The sample was oxidized to convert all C to CO_2_ and passed through the NDIR detector to quantify TC concentration. Inorganic C (carbonated and dissolved CO_2_) was analyzed by acidifying samples to a pH less than 3 using phosphoric acid, conversion to CO_2_, and measured on the NDIR detector. Total OC was calculated by subtraction of IC from TC. Total N was determined by the sample being sent through the Shimadzu TOC-L and decomposed at 720 ^o^C to NO then detected by chemiluminescence at 50 ^o^C using the Shimadzu TNM-L instrument (Shimadzu Scientific Instruments, Columbia, MD). Nitrate N in the PL extracts was analyzed using QuickChem Method 10-107-04-1-B on a Lachat QuickChem FIA+ 8000 instrument (Hach Company, Loveland, CO). Briefly, nitrate in the sample is reduced to nitrite by passage through a copperized cadmium column, diazotized with sulfanilamide, coupled with N-(1-naphthyl) ethylenediamine dihydrochloride, and absorbance is measured at 520 nm. The Lachat QuickChem instrument was also used to analyze for NH_4_-N using Quickchem Method 12-107-06-2-A where the sample is heated at 60 ^o^C with salicylate and hypochlorite in an alakaline phosphate buffer followed by measuring the absorbance of the reaction product at 660 nm. Water soluble cations were measured directly in the filtered PL extract samples using an Agilent 5110 VDV inductively coupled plasma (ICP) spectrophotometer (Agilent Technologies, Santa Clara, CA).

**Poultry litter extraction.** We prepared a filter-sterilized slurry from the PL used in this study. A 1:10 PL slurry was made in autoclaved 1X Phosphate Buffer Saline (PBS) (Fisher Sci, Hampton, NH) and homogenized for 1 h at 450 rpm in a hand wrist shaker (Boekel Scientific, Feasterville, PA). Afterwards, PL slurry was centrifuged at 4,600 x *g* for 30 min. Supernatant was sequentially filtered through 1.2 μm, 0.45 μm and 0.2 μm pore –sized polycarbonate membrane filters; and hereafter termed “poultry litter extract” (PLE). The absence of bacteria was confirmed by culturing 100 μl of PLE in Brain Heart Infusion broth (BHIB).

**Genomic and plasmid DNA preparation.** *S*. Heidelberg strain SH-116 was used to prepare standard curves for chromosomal and plasmid encoded genes. Briefly, archived pure cultures were streaked onto SBA and incubated overnight at 37 ^o^C for 24 h. Following overnight growth, 5 – 6 single colonies were randomly chosen and resuspended in 200 µl molecular grade DEPC water. Genomic DNA was extracted from resuspended cells with FastDNA Spin Kit (Mp Biomedicals, Solon, Ohio) according to manufacturer’s instructions. For plasmid DNA, a 100 µl pipet tip was stabbed into frozen archived pure culture and propagated overnight in 10 ml of LB broth. After propagation, 400 µl of starter culture was transferred to fresh 40 ml LB broth and incubated in a water bath shaker at 37 ^o^C and 240 rpm for 3 - 4 h. Bacterial culture was harvested by centrifugation at 4,700 x *g* for 15 min. Plasmid DNA was extracted from bacterial pellet with Qiagen Plasmid Midi kit (Qiagen Inc, Germantown, MD) as per manufacturer’s instructions. DNA extracted was quantified fluorometrically using Qubit Fluorometer (ThermoFisher Scientific). DNA was stored at -80 ^o^C until use.

**qPCR assays and quantification.** Primers used in this study are shown in S9 Table. Unless otherwise stated, primers were designed with beacon designer (Premier Biosoft, Palo Alto, CA) and synthesized by Integrated DNA Technologies (Coralville, IA). Primers were rehydrated to a concentration of 100 µM in 1X TE buffer. qPCR assays were performed with CFX96 Touch Real-Time PCR Detection System (Bio-Rad Inc., Hercules, CA). Reaction mixtures (20 µl) for all assays contained 1X SsoAdvanced Universal SYBR Green Supermix (Bio-Rad Inc., Hercules, CA), 0.02 mg/ml bovine serum albumin (ThermoFisher Scientific), 250nM (each) primers, and 2 µl of either genomic DNA (PL samples or pure cultures), 10^3^ to 10^8^ plasmid gene copies or 200 to 2 X 10^7^ chromosome gene copies. All reactions were duplicated in Hard-Shell 96-well PCR reaction plates (Bio-Rad Inc., Hercules, CA) covered with MicroAmp optical adhesive film (ThermoFisher Scientific). Thermal conditions for all assays were 98 °C for 3 min (initial denaturation), followed by 40 cycles of short denaturation at 95 °C for 15 s and a combined annealing and primer extension phase at 60 °C for 30 s. A melt curve was performed on every assay and sample. Data were analyzed with Bio-Rad CFX manager and baseline and threshold were determined automatically. Threshold cycle (C_T_) values were exported to Microsoft Excel for further statistical analysis. To prevent cross-contamination, dedicated equipment and separate laboratories were used for every step from DNA extraction to qPCR amplification. In addition, genomic or plasmid standards representative of our range of quantification were included for each assay performed in a 96-well qPCR plate.

**Determination of plasmid-specific genes in different sources of poultry litter.** To assess the prevalence of Col plasmids in PL, we determined the concentration of plasmid-specific genes in various litter collected for another on-going experiment in our laboratory. These litter were sourced from 7 poultry farms in 4 states in USA, practicing either conventional or backyard method of raising chicken flocks. DNA was extracted in triplicates from each PL using Qiagen DNeasy Power-Soil. We performed qPCR on 2 µl of purified DNA extract as described previously in qPCR assays, with primer sets specific to each plasmid of interest (S9 Table).

**References:**

1. Orlek A, Phan H, Sheppard AE, Doumith M, Ellington M, Peto T, et al. Ordering the mob: Insights into replicon and MOB typing schemes from analysis of a curated dataset of publicly available plasmids. Plasmid. 2017;91:42-52.

2. Altschul SF, Madden TL, Schaffer AA, Zhang J, Zhang Z, Miller W, et al. Gapped BLAST and PSI-BLAST: a new generation of protein database search programs. Nucleic Acids Res. 1997;25(17):3389-402.

3. Orlek A, Phan H, Sheppard AE, Doumith M, Ellington M, Peto T, et al. A curated dataset of complete Enterobacteriaceae plasmids compiled from the NCBI nucleotide database. Data Brief. 2017;12:423-6.

4. Prjibelski AD, Vasilinetc I, Bankevich A, Gurevich A, Krivosheeva T, Nurk S, et al. ExSPAnder: a universal repeat resolver for DNA fragment assembly. Bioinformatics. 2014;30(12):i293-301.

5. Wick RR, Schultz MB, Zobel J, Holt KE. Bandage: interactive visualization of de novo genome assemblies. Bioinformatics. 2015;31(20):3350-2.

6. Wick RR, Judd LM, Gorrie CL, Holt KE. Unicycler: Resolving bacterial genome assemblies from short and long sequencing reads. PLoS Comput Biol. 2017;13(6):e1005595.

7. Katoh K, Standley DM. MAFFT multiple sequence alignment software version 7: improvements in performance and usability. Mol Biol Evol. 2013;30(4):772-80.

8. Kumar S, Stecher G, Tamura K. MEGA7: Molecular Evolutionary Genetics Analysis Version 7.0 for Bigger Datasets. Mol Biol Evol. 2016;33(7):1870-4.
