# Supplementary figures and images for: Hotspot mutations and ColE1 plasmids contribute to the fitness of Salmonella Heidelberg in poultry litter
Source: PLoS One. 2018 Aug 31;13(8):e0202286. doi: 10.1371/journal.pone.0202286 (PMC6118388; doi:10.1371/journal.pone.0202286)

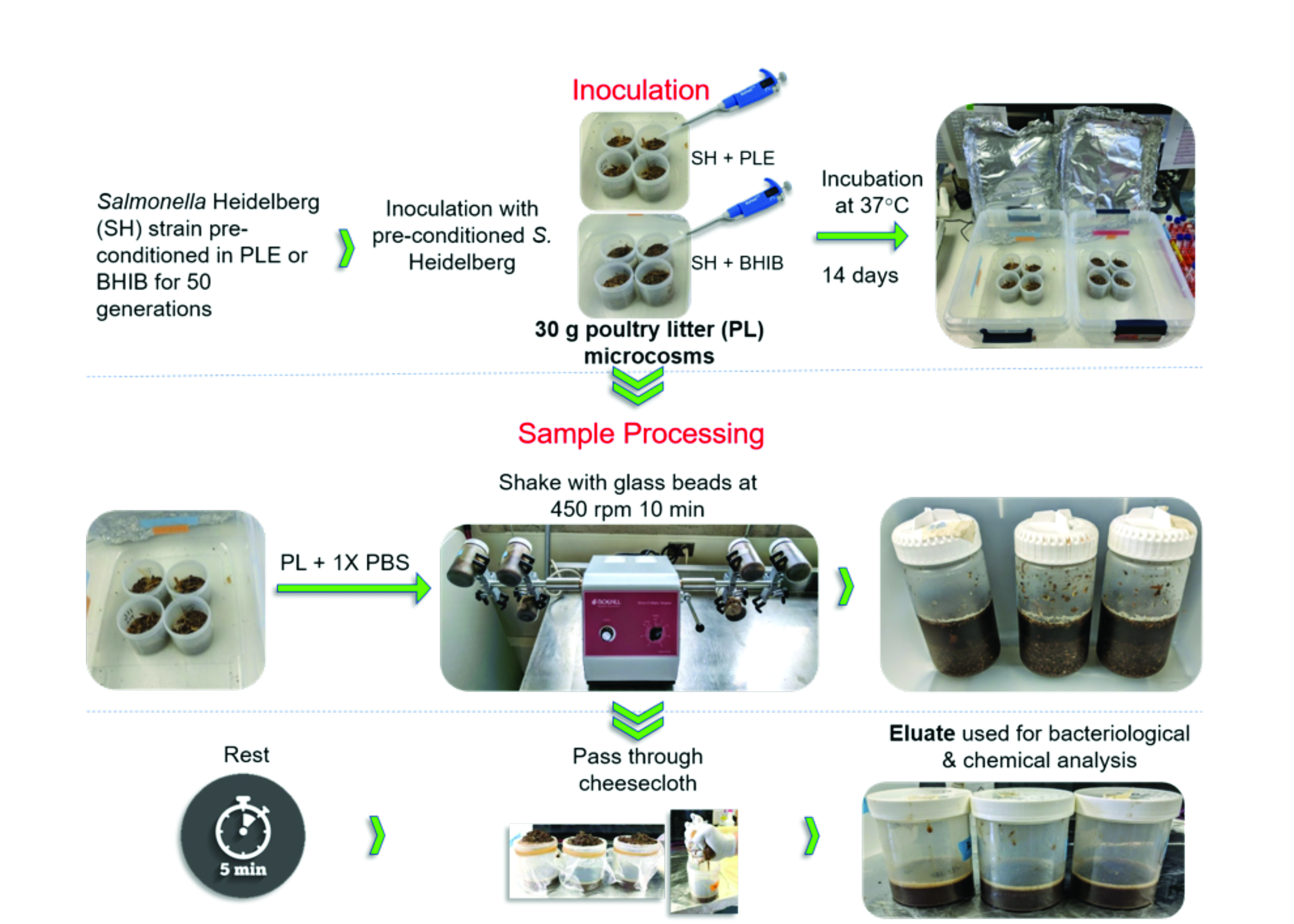

Supplement: S1 Fig — Forty-eight “used” poultry litter microcosms were individually inoculated with either SH-2813 (n = 24) or SH-116 (n = 24) preconditioned in either BHIB (n = 12) or PLE (n = 12) before incubation at 37°C for 14 days. (TIF) [file pone.0202286.s002.tif]

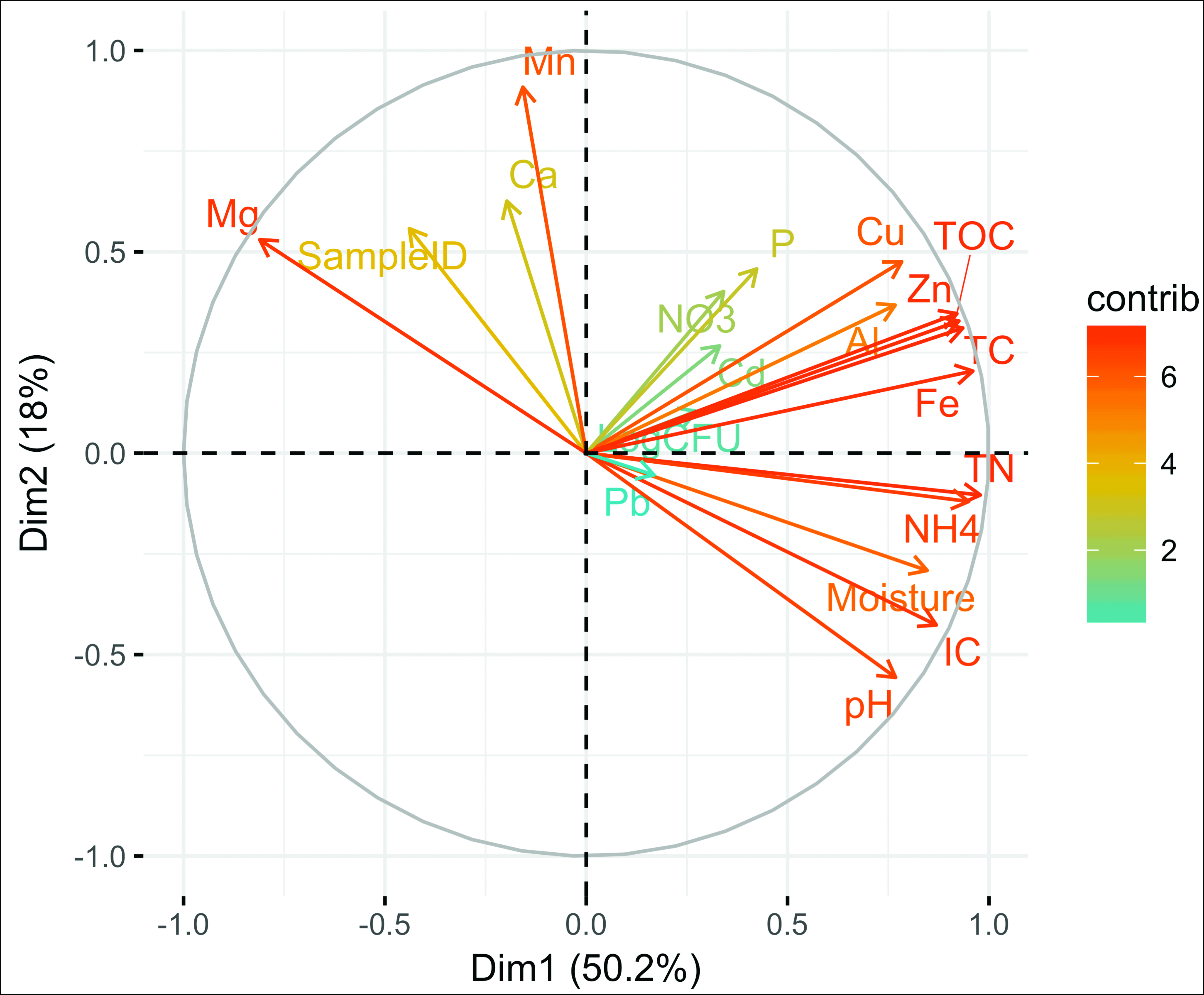

Supplement: S2 Fig — Data represent 48 microcosms. Positively correlated variables point to the same side of the plot and negative correlated variables point to opposite sides of the graph. Symbol: Colored by contributions to the principal component (Contrib). (TIF) [file pone.0202286.s003.tif]

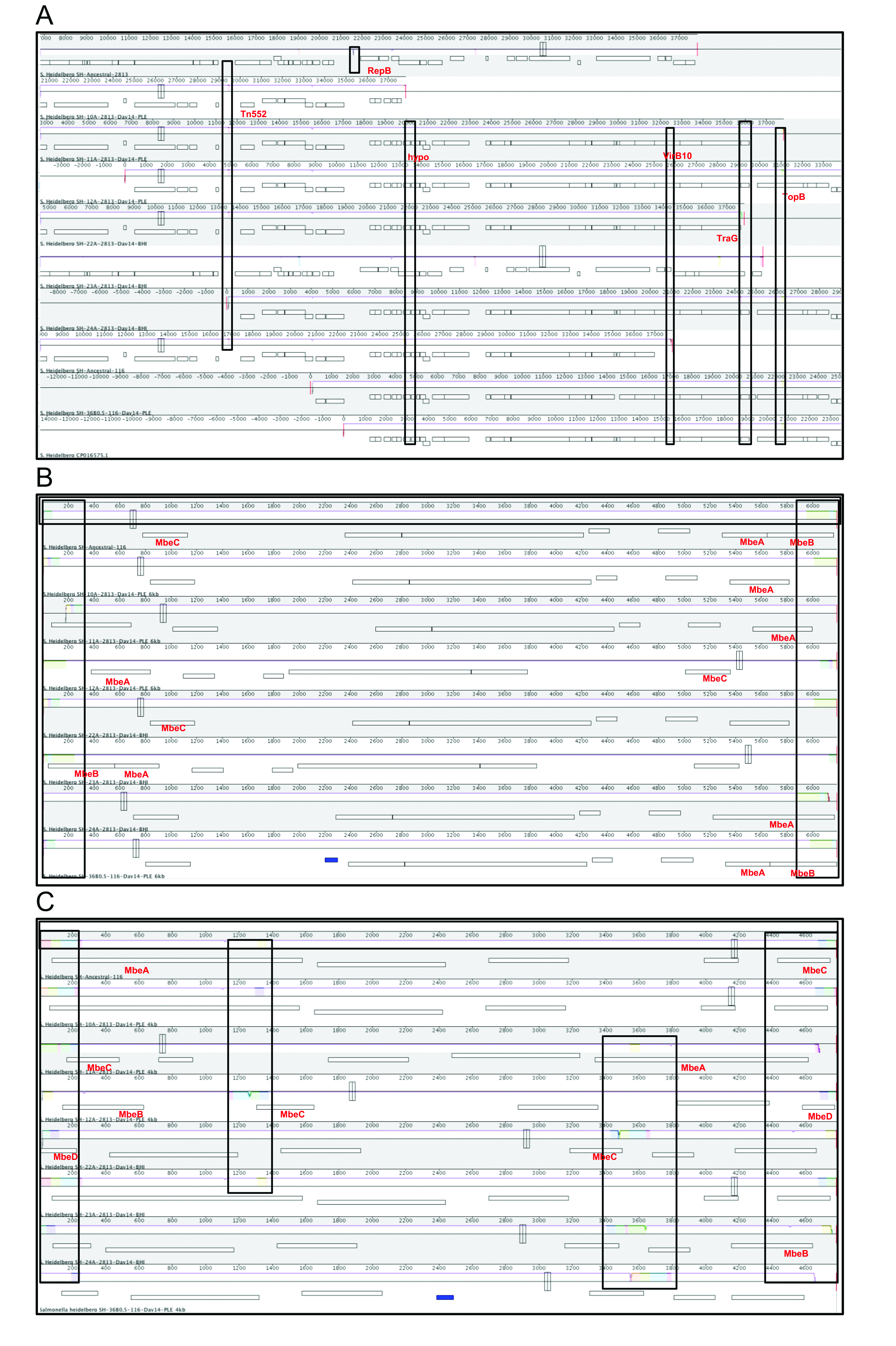

Supplement: S3 Fig — ProgressiveMauve alignment of (a) IncX1 (b) ColE1-6kb and (c) ColE1-4kb whole plasmid DNA sequence from selected isolates from this study. DNA regions that differ are highlighted with horizontal black rectangular boxes. (TIF) [file pone.0202286.s004.tif]

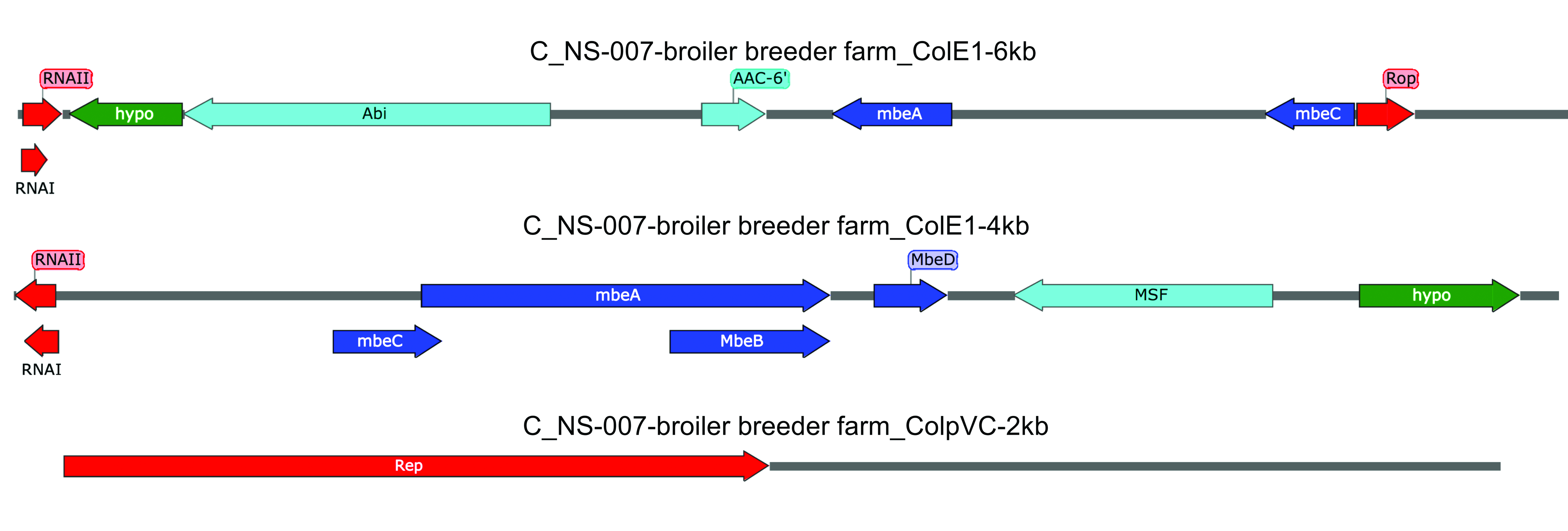

Supplement: S4 Fig — Strain NS-007 (NCBI accession: SAMN08031253) was isolated from a broiler breeder farm in the USA under Bioproject PRJNA417775. Contig size for ColE1-6kb, ColE1-4kb and ColpVC-2kb are 6183, 4778 and 2105 bp, respectively. (TIF) [file pone.0202286.s005.tif]

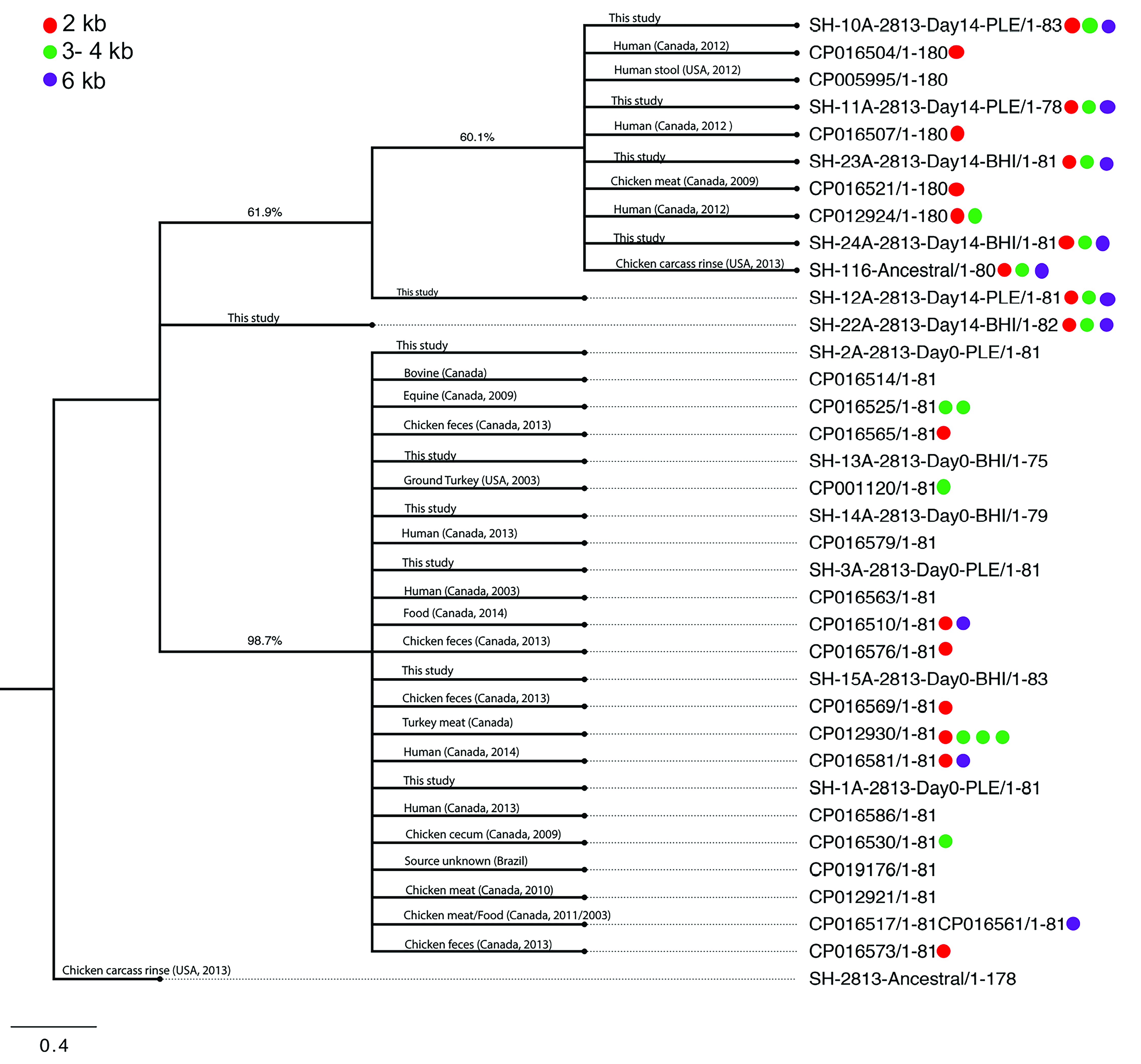

Supplement: S6 Fig — The consensus 23S-5S ITS sequence of isolates included from this study were extracted by mapping whole genome sequence reads to S. Heidelberg reference genome (CP016573). Symbols: red, green and blue circles next to taxa name denotes Col-like plasmids present in sequenced genome. (TIF) [file pone.0202286.s007.tif]

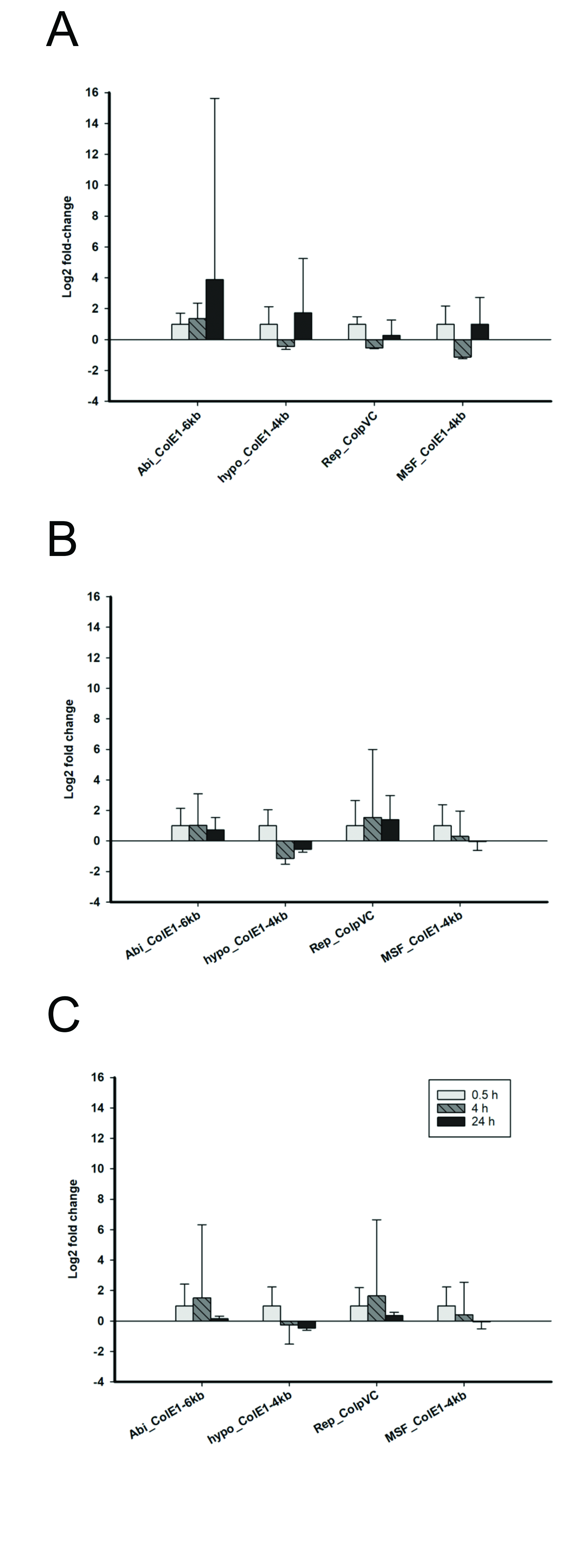

Supplement: S7 Fig — Gene expression of selected Col plasmid genes determined by qRT-PCR for (a) SH-116anc (b) SH-116evol and (c) SH-2813evol isolates. Log2 fold-change is relative to the expression of chromosome encoded reference genes gapA and gyrB. Symbols: replication protein of ColpVC (ColpVC-rep), abortive phage infection protein of ColE1-6kb (ColE1-6kb-Abi), macrophage stimulating factor protein of ColE1-4kb (ColE1-4kb-MSF) and hypothetical protein (hypo). (TIF) [file pone.0202286.s008.tif]

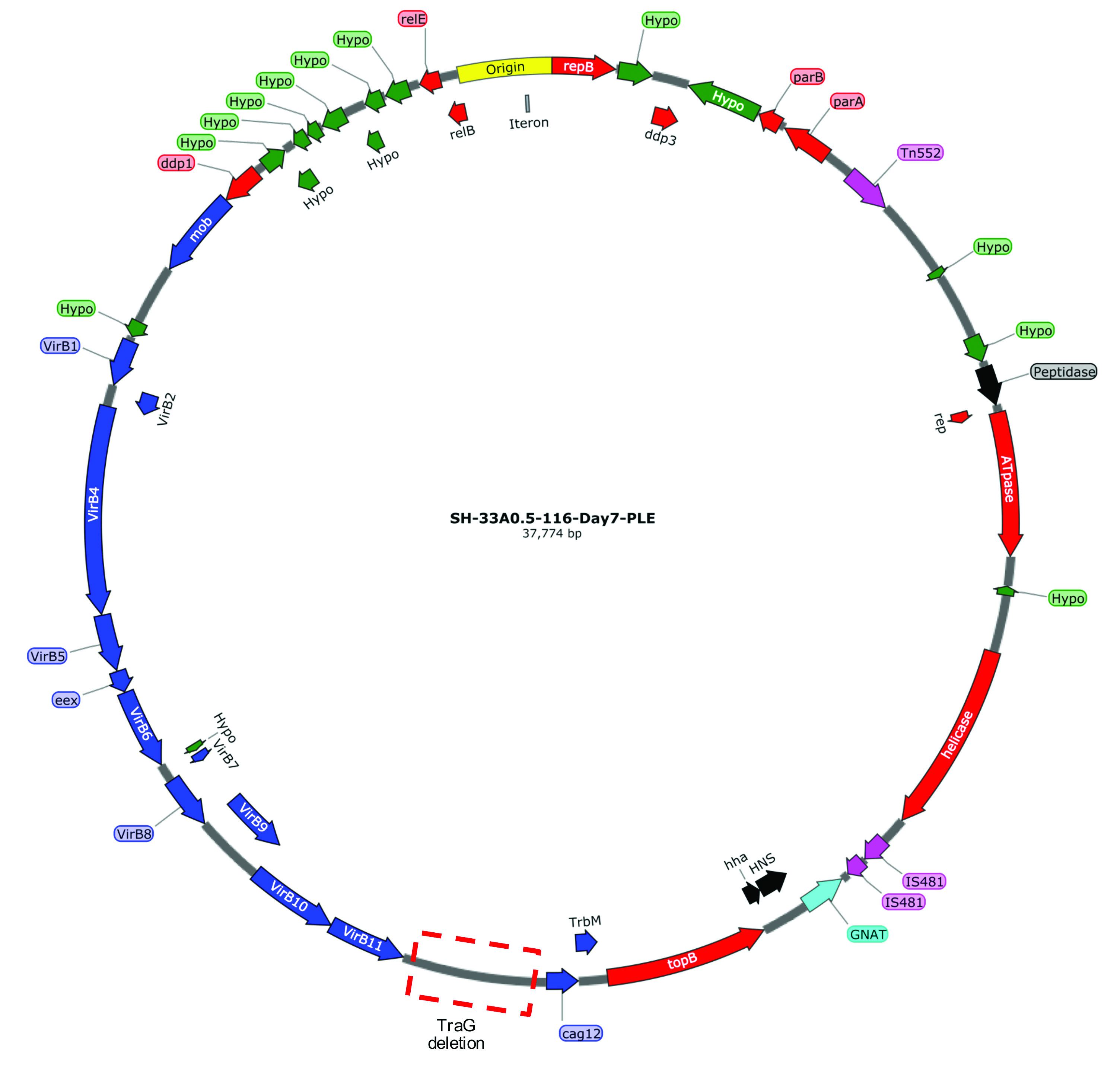

Supplement: S8 Fig — (TIF) [file pone.0202286.s009.tif]
