# Supplementary material for: Hotspot mutations and ColE1 plasmids contribute to the fitness of Salmonella Heidelberg in poultry litter
Source: PLoS One. 2018 Aug 31;13(8):e0202286. doi: 10.1371/journal.pone.0202286 (PMC6118388; doi:10.1371/journal.pone.0202286)
Supplement: S5 Fig — (PDF) [file pone.0202286.s006.pdf]

Isolate ID	Gifsy_2	PaV_LD	Sal4
**SH-Ancestral-2813**			
SH-1A-2813-Day0-PLE			
SH-2A-2813-Day0-PLE			
SH-3A-2813-Day0-PLE			
SH-4A-2813-Day1-PLE			
SH-4B-2813-Day1-PLE			
SH-5A-2813-Day1-PLE			
SH-5B-2813-Day1-PLE			
SH-6A-2813-Day1-PLE			
SH-6B-2813-Day1-PLE			
SH-7A-2813-Day7-PLE			
SH-7B-2813-Day7-PLE			
SH-8A0.5-2813-Day7-PLE			
SH-8A-2813-Day7-PLE			
SH-8B-2813-Day7-PLE			
SH-9A-2813-Day7-PLE			
SH-9B-2813-Day7-PLE			
SH-10A-2813-Day14-PLE			
SH-10B-2813-Day14-PLE			
SH-11A-2813-Day14-PLE			
SH-11B-2813-Day14-PLE			
SH-12A-2813-Day14-PLE			
SH-12B-2813-Day14-PLE			
SH-13A-2813-Day0-BHI			
SH-13B-2813-Day0-BHI			
SH-14A-2813-Day0-BHI			
SH-14B-2813-Day0-BHI			
SH-15A-2813-Day0-BHI			
SH-15B-2813-Day0-BHI			
SH-16ABPW-2813-Day1-BHI			
SH-16BBPW-2813-Day1-BHI			
SH-17ABPW-2813-Day1-BHI			
SH-17BBPW-2813-Day1-BHI			
SH-18ABPW-2813-Day1-BHI			
SH-18BBPW-2813-Day1-BHI			
SH-19A0.5-2813-Day7-BHI			
SH-19A-2813-Day7-BHI			
SH-19B-2813-Day7-BHI			

SH-20A-2813-Day7-BHI			
SH-20B-2813-Day7-BHI			
SH-21A-2813-Day7-BHI			
SH-21B-2813-Day7-BHI			
SH-22A0.5-2813-Day14-BHI			
SH-22A-2813-Day14-BHI			
SH-22B-2813-Day14-BHI			
SH-23A-2813-Day14-BHI			
SH-23B-2813-Day14-BHI			
SH-24A-2813-Day14-BHI			
SH-24B-2813-Day14-BHI			
**SH-Ancestral-116**			
SH-25A-116-Day0-PLE			
SH-25B-116-Day0-PLE			
SH-26A-116-Day0-PLE			
SH-26B-116-Day0-PLE			
SH-27A-116-Day0-PLE			
SH-27B-116-Day0-PLE			
SH-28B-116-Day1-PLE			
SH-29B-116-Day1-PLE			
SH-30B-116-Day1-PLE			
SH-31A0.5-116-Day7-PLE			
SH-31A-116-Day7-PLE			
SH-31B-116-Day7-PLE			
SH-32A-116-Day7-PLE			
SH-32B-116-Day7-PLE			
SH-33A0.5-116-Day7-PLE			
SH-33A-116-Day7-PLE			
SH-33B-116-Day7-PLE			
SH-36A0.5-116-Day14-PLE			
SH-36B0.5-116-Day14-PLE			
SH-37A-116-Day0-BHI			
SH-37B-116-Day0-BHI			
SH-38A-116-Day0-BHI			
SH-38B-116-Day0-BHI			
SH-39A-116-Day0-BHI			
SH-39B-116-Day0-BHI			
SH-40BBPW-116-Day1-BHI			
SH-41BBPW-116-Day1-BHI			
SH-42BBPW-116-Day1-BHI			

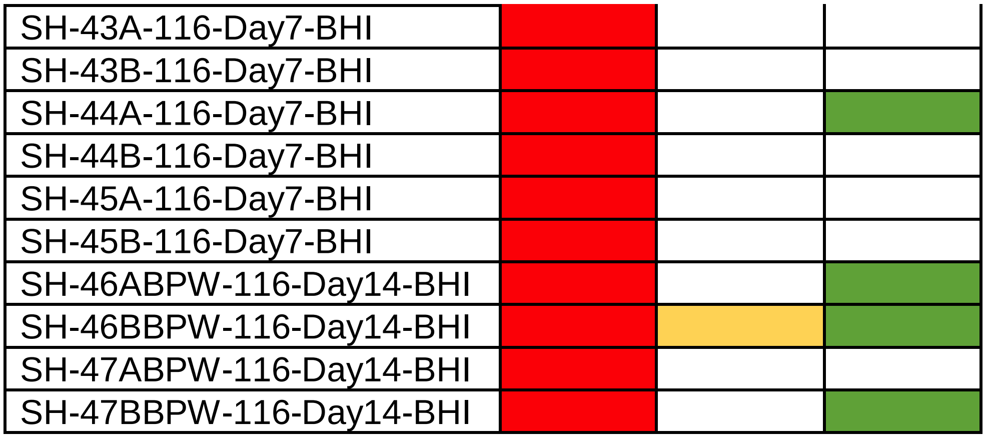

Fig. S5. Intact prophages in sequenced genomes of *S*. Heidelberg from this study. Symbols: red cells – intact Gifsy-2 prophage (NC_010393), yellow cells – intact PaV-LD (NC_016564) prophage, green cells – intact sal-4 (NC_030919) prophage.
