# Supplementary material for: Hotspot mutations and ColE1 plasmids contribute to the fitness of Salmonella Heidelberg in poultry litter
Source: PLoS One. 2018 Aug 31;13(8):e0202286. doi: 10.1371/journal.pone.0202286 (PMC6118388; doi:10.1371/journal.pone.0202286)
Supplement: S1 Table — (DOCX) [file pone.0202286.s010.docx]

|  |  | Bacteria concentration (Log CFU/ g dry Weight)  Mean (S.E) | | |
| --- | --- | --- | --- | --- |
| Treatments/Microcosms | Day | *Salmonella* | Total aerobic bacteria | Enterococci |
| SH-2813-PLE | 0 | 6.10 (0.02) | 7.82 (0.02) | 4.79 (0.06) |
|  | 1 | 1.91 (0.05) | 6.2 (0.12) | 4.92 (0.09) |
|  | 7 | 3.43 (0.22) | 7.33 (0.06) | 3.39 (0.29) |
|  | 14 | 2.47 (0.03) | 4.71 (0.27) | 3.66 (0.08) |
|  |  |  |  |  |
| SH-2813-BHIB | 0 | 5.42 (0.06) | 7.36 (0.69) | 4.45 (0.27) |
|  | 1 | <LOQ = 7 (0.04)* | 5.40 (0.19) | 4.00 (0.44) |
|  | 7 | 3.45 (0.04) | 7.59 (0.02) | 3.90 (0.76) |
|  | 14 | 2.51 (0.05) | 5.00 (0.05) | 3.32 (0.03) |
|  |  |  |  |  |
| SH-116-PLE | 0 | 5.64 (0.06) | 6.28 (0.03) | 4.47 (0.03) |
|  | 1 | 2.81 (0.18) | 6.58 (0.43) | 4.59 (0.19) |
|  | 7 | 2.33 (0.50) | 6.88 (0.06) | 4.17 (0.04) |
|  | 14 | <LOQ = 2.83 (2.13)* | 3.62 (0.16) | 3.49 (0.11) |
|  |  |  |  |  |
| SH-116-BHIB | 0 | 5.81(0.14) | 6.30 (0.16) | 4.21 (0.24) |
|  | 1 | <LOQ = 7 (0.09)* | 6.06 (0.53) | 3.53 (0.12) |
|  | 7 | 2.29 (0.22) | 6.91 (0.08) | 4.21 (0.26) |
|  | 14 | <LOQ = 2.88 (2.02)* | 4.32 (0.00) | 3.02 (0.03) |

Note. – PLE – Poultry Litter Extract; BHIB – Brain Heart Infusion Broth.

Limit of quantification (LOQ) for direct quantification of *Salmonella* from poultry litter was 10 CFU/g dry weight.

^*^Asterisk = Detection of bacteria was only possible after enrichment in Buffered Peptone Water (BPW). Number reported is concentration following overnight enrichment in BPW
