# Supplementary material for: Hotspot mutations and ColE1 plasmids contribute to the fitness of Salmonella Heidelberg in poultry litter
Source: PLoS One. 2018 Aug 31;13(8):e0202286. doi: 10.1371/journal.pone.0202286 (PMC6118388; doi:10.1371/journal.pone.0202286)
Supplement: S2 Table — (DOCX) [file pone.0202286.s011.docx]

| **Strain** | **Treatment** | **Day** | **pH** | **Moisture** | **NH_4_** | **NO_3_** | **Al** | **Ca** | **Cd** | **Mg** | **Mn** | **P** | **Pb** | **Zn** |
| --- | --- | --- | --- | --- | --- | --- | --- | --- | --- | --- | --- | --- | --- | --- |
|  |  |  |  |  |  |  |  |  |  |  |  |  |  |  |
|  |  |  |  |  | ----------------------------------------------------------------------------------------------mg/kg------------------------------------------------------------------------------------------------------------ | | | | | | | | | |
|  | PLE | 0 | 7.96 ± 0.05 | 0.26 ± 0.01 | 7640.09 ± 354.80 | 692.81 ± 40.71 | 12.28 ± 0.22 | 1120.54 ± 41.91 | 0.47 ± 0.07 | 531.10 ± 34.35 | 54.58 ± 2.54 | 17055.90 ± 884.75 | 46.37 ± 6.99 | 423.60 ± 10.41 |
| SH-2813 |  | 14 | 7.92 ± 0.01 | 0.12 ± 0.00 | 4156.27 ± 216.71 | 973.42 ± 275.57 | 7.85 ± 0.59 | 1102.11 ± 70.27 | 0.69 ± 0.13 | 782.91 ± 51.89 | 63.05 ± 3.34 | 16172.70 ± 841.12 | 33.51 ± 10.15 | 371.52 ± 20.98 |
|  | BHIB | 0 | 8.86 ± 0.01 | 0.35 ± 0.01 | 19777.40 ± 583.25 | 1214.52 ± 233.42 | 13.31 ± 0.52 | 781.94 ± 33.96 | 0.81 ± 0.04 | 82.65 ± 6.94 | 43.55 ± 2.01 | 17036.10 ± 767.84 | 55.03 ± 8.47 | 496.74 ± 20.99 |
|  |  | 14 | 8.29 ± 0.02 | 0.18 ± 0.01 | 3131.96 ± 372.75 | 883.27 ± 314.42 | 5.78 ± 1.02 | 947.65 ± 125.87 | 0.57 ± 0.08 | 460.66 ± 37.67 | 49.46 ± 6.61 | 17469.70 ± 574.66 | 55.26 ± 2.24 | 338.97 ± 59.89 |
|  | PLE | 0 | 8.21 ± 0.03 | 0.32 ± 0.01 | 9120.39 ± 122.20 | 929.55 ± 320.90 | 12.26 ± 0.42 | 1003.79 ± 31.87 | 1.33 ± 0.18 | 334.44 ± 41.25 | 53.51 ± 0.87 | 16786.20 ± 328.17 | 44.24 ± 15.90 | 443.17 ± 5.74 |
| SH-116 |  | 14 | 8.01 ± 0.02 | 0.17 ± 0.01 | 5016.90 ± 108.53 | 1041.40 ± 354.11 | 8.14 ± 0.46 | 902.10 ± 51.51 | 0.47 ± 0.10 | 453.94 ± 14.19 | 49.40 ± 2.08 | 15983.90 ± 495.79 | 48.53 ± 2.79 | 361.27 ± 13.46 |
|  | BHIB | 0 | 8.90 ± 0.02 | 0.37 ± 0.01 | 17589.90 ± 344.15 | 965.23 ± 154.78 | 12.45 ± 0.54 | 836.17 ± 41.22 | 0.61 ± 0.17 | 83.54 ± 17.70 | 43.41 ± 0.81 | 16213.80 ± 309.21 | 69.10 ± 11.03 | 501.53 ± 11.03 |
|  |  | 14 | 8.29 ± 0.04 | 0.19 ± 0.01 | 4097.35 ± 329.14 | 563.94 ± 59.81 | 6.54 ± 0.39 | 1022.67 ± 13.19 | 0.69 ± 0.31 | 399.21 ± 27.76 | 49.89 ± 3.38 | 15900.03 ± 696.43 | 33.08 ± 2.93 | 352.54 ± 20.65 |

Note. – PLE – Poultry Litter Extract; BHIB – Brain Heart Infusion Broth
