# Supplementary material for: Hotspot mutations and ColE1 plasmids contribute to the fitness of Salmonella Heidelberg in poultry litter
Source: PLoS One. 2018 Aug 31;13(8):e0202286. doi: 10.1371/journal.pone.0202286 (PMC6118388; doi:10.1371/journal.pone.0202286)
Supplement: S9 Table — (DOCX) [file pone.0202286.s018.docx]

| **Assay name** | **Primer** | **Primer sequence (5 '- 3')** | **Target Gene** | **Genome** | **Amplification factor** | **Melt curve (T_m_ )** | **Reference** |
| --- | --- | --- | --- | --- | --- | --- | --- |
| 16S rRNA* | F | CCTCTTGCCATCGGATGTG | 16S Ribosomal RNA | Chromosome | 1.75 | 84 °C | (8) |
|  | R | GGCTGGTCATCCTCTCAGACC |  |  |  |  |  |
| *gyrB* | F | ATAGAAGAAGGTCAACAG | Gyrase Beta Subunit | Chromosome | 2.02 | 84 °C | This study |
|  | R | TATCACAGCATCATCATC |  |  |  |  |  |
| *gapA* | F | TTGGAGATGTGAGCAATC | Glyceraldehyde-6-dehydrogenase | Chromosome | 1.93 | 80.5 °C | This study |
|  | R | GACAACTTCGTGAAACTG |  |  |  |  |  |
| repB_IncX1 | F | TGGACATACGAAGAAGAG | Pir Family of Replicase | IncX1 plasmid | 1.90 | 79.5 °C | This study |
|  | R | AACCTGAGTAGTGTAAGAAT |  |  |  |  |  |
| *relB* | F | GCTGAAGATGCTGAGTTA | Type II Antitoxin | IncX1 plasmid | 1.96 | 81 °C | This study |
|  | R | TCATAGGTCATCCAGGTTA |  |  |  |  |  |
| *relE* | F | GTCATGCTGATCGCTATA | Type II Toxin | IncX1 plasmid | 1.90 | 80 °C | This study |
|  | R | AACGGCAATAACGAGTAA |  |  |  |  |  |
| MSF_ColE1-4kb | F | GTTCCTAATACAGTGATTCCT | Putative Macrophage Stimulating Factor | ColE1-6kb | 1.99 | 79.5 °C | This study |
|  | R | TTGCGTTGCTAATATCCTT |  |  |  |  |  |
| AAC-(6')_ColE1-6kb | F | CTTTTCACTCGGTATCTG | Putative aminoglycoside-6-N-acetyltransferase | ColE1-6kb | 1.95 | 81 °C | This study |
|  | R | TCATGTTTCACTCCCTAC |  |  |  |  |  |
| Abi_ColE1-6kb | F | GCGAGATGGTCAACTTAT | Putative Type IV Toxin-Antitoxin System | ColE1-4kb | 1.96 | 79 °C | This study |
|  | R | AATCCAGCAACAACAGAA |  |  |  |  |  |
| rep_ColpVC | F | ATGGTGATCGGTGATGAT | Replication Protein | ColpVC-2kb | 2.03 | 83 °C | This study |
|  | R | TACTTTCTGACCTCGGTTT |  |  |  |  |  |
| ColRNAI-ColE1-6kb | F | ATGGTGATCGGTGATGAT | IncRNA | ColE1-6kb | 1.93 | 83 °C | This study |
|  | R | TACTTTCTGACCTCGGTTT |  |  |  |  |  |

| ColRNAI-ColE1-4kb | F | ATGGTGATCGGTGATGAT | IncRNA | ColE1-4kb | 1.89 | 81 °C | This study |
| --- | --- | --- | --- | --- | --- | --- | --- |
|  | R | TACTTTCTGACCTCGGTTT |  |  |  |  |  |

Note. – F- Forward primer, R - Reverse primer

^*^16S rRNA primers targeting the family Enterobacteriaceae
